# Host Adaptation and the Alteration of Viral Properties of the First Influenza A/H1N1pdm09 Virus Isolated in Japan

**DOI:** 10.1371/journal.pone.0130208

**Published:** 2015-06-16

**Authors:** Akira Ainai, Hideki Hasegawa, Masatsugu Obuchi, Takato Odagiri, Makoto Ujike, Masayuki Shirakura, Eri Nobusawa, Masato Tashiro, Hideki Asanuma

**Affiliations:** 1 Influenza Virus Research Center, National Institute of Infectious Diseases, Gakuen 4-7-1, Musashi-Murayama-shi, Tokyo, 208–0011, Japan; 2 Department of Pathology, National Institute of Infectious Diseases, Toyama 1-23-1, Shinjuku-ku, Tokyo, 162–8640, Japan; 3 Department of Veterinary Medicine, Nippon Veterinary and Life Science University, Kyonancho 1-7-1, Musashino-shi, Tokyo, 180–8602, Japan; University of Geneva, SWITZERLAND

## Abstract

A/Narita/1/2009 (A/N) was the first H1N1 virus from the 2009 pandemic (H1pdm) to be isolated in Japan. To better understand and predict the possible development of this virus strain, the effect of passaging A/N was investigated in Madin-Darby canine kidney cells, chicken eggs and mice. A/N that had been continuously passaged in cells, eggs, or mice obtained the ability to grow efficiently in each host. Moreover, A/N grown in mice had both a high level of pathogenicity in mice and an increased growth rate in cells and eggs. Changes in growth and pathogenicity were accompanied by amino acid substitutions in viral hemagglutinin (HA) and PB2. In addition, the adapted viruses exhibited a reduced ability to react with ferret antisera against A/N. In conclusion, prolonged passaging allowed influenza A/N to adapt to different hosts, as indicated by a high increase in proliferative capacity that was accompanied by an antigenic alteration leading to amino acid substitutions.

## Introduction

In March 2009, a new influenza virus of swine origin, A/H1N1pdm09 (H1pdm), emerged in Mexico and the USA. The virus quickly spread worldwide through human-to-human transmission. In response to the number of countries and communities reporting human cases, the World Health Organization raised the influenza pandemic alert to the highest level (level 6) on June 11, 2009.

Genomic analyses were performed on the H1pdm virus as soon as it began to spread rapidly in California and Mexico, and results revealed that it originated from several viruses that had circulated in pigs for a number of years, namely the North American H3N2 triple-reassortant, the classical swine H1N1 lineage and the Eurasian ‘avian-like’ swine H1N1 virus [1–3]. Sequencing of virus isolates revealed the absence of markers associated with a high level of pathogenicity in avian and mammalian H5N1 species such as a multibasic hemagglutinin (HA) cleavage site [4] or lysine at position 627 of the PB2 protein [5,6]. This agreed with findings that the newly developed H1N1 virus was not highly pathogenic in humans.

The prompt production of a vaccine for H1pdm was deemed a global priority in light of the rapid worldwide spread of the pandemic. However, the H1pdm virus was not adapted to growth in eggs. Therefore, in the early stages of the epidemic, classical reassorting processes were used to develop high-growth reassortant vaccine strains X-179A and IVR-153 at the New York Medical College and CSL, Australia. These first vaccine candidates were useful but the yield was only approximately half the amount of antigen typically obtained for a seasonal H1N1 virus [7,8]. Currently, cell culture-derived technology is regarded as a better option for the manufacture of large vaccine quantities in the event of a shortage. In addition, this technology offers the potential to elicit an improved immune response through seed viruses that closely match the wild virus and also provides an option for people who are allergic to eggs [9]. Two cell-based H1pdm vaccines have been licensed and are currently used, Gelture and Optaflu [10]. Unfortunately, substitutions at residues 153–157 within HA often emerge upon passaging of the virus in cell cultures, and these substitutions are associated with reduced titers in hemagglutination inhibition (HAI) assays [11].

In Japan, the first cases of swine flu were confirmed on May 9, 2009 in three people who arrived in Tokyo from Canada [12]. The patients had influenza-like symptoms and tested positive according to a primary diagnostic test. The diagnosis was confirmed with an H1pdm-specific quantitative PCR test performed at the National Institute of Infectious Diseases. The patients were hospitalized in Tokyo near the airport and all three subsequently recovered. The influenza strain isolated from one of three patients, A/Narita/1/2009 (A/N), was the first H1pdm strain to be isolated in Japan. The sequence of this strain was consistent with most of the strains isolated early in the pandemic, so the three patients were suspected of having been infected during their visit to Canada. Although the A/N strain was the first to be isolated in Japan after the onset of the epidemic and this strain was subsequently used as an experimental standard, there is currently no information available regarding its ability to change after adaptating to different hosts. Therefore, the current study investigated the effect that passaging A/N in Madin-Darby canine kidney (MDCK) cells, chicken eggs, and mice had on virus infectivity, pathogenicity, and antigenicity.

## Materials and Methods

### Animals

Ferrets (6 to 12-month-old, female) and mice (6 to 8-week-old, female) were purchaced from Japan SLC (City of Hamamatsu, Shizuoka Pref, Japan) and used in this study. All animal experiments were performed under animal biosafety level 3 conditions. Groups of two ferrets were used to prepare antisera against cell or egg isolates of A/Narita/1/2009 viruses (A/N) in order to evaluate antigenicity. One antiserum for each virus was used because the two ferrets had the same titer. Mice were used to establish mouse-adapted virus and to estimate the lethal dose in mice. All animal experiments were performed in accordance with the Guidelines for Animal Experiments Performed at the National Institute of Infectious Diseases (NIID) and were approved by the Animal Care and Use Committee of the NIID (approval No. 109037 & 109049). Virus inoculation and euthanasia were performed under anesthesia with ketamine and xylazine, and all efforts were made to minimize suffering.

### Virus passages in cells and eggs

Egg- or MDCK cell-isolated viruses A/N were used in this study and the accession numbers of each genome segment of A/N isolates are shown in Table 1. Cell-isolated A/N was diluted 10^3^-fold with virus isolation medium for subsequent culture in 6-well plates containing 2 mL of virus solution per well. When CPE were observed, all 6 wells of a culture plate were pooled and used in subsequent passages. Egg-isolated A/N was diluted 10^3^-fold with phosphate buffered saline (PBS) and 0.2 mL of virus suspension was inoculated into eggs. After each passage from passages 2–15 in eggs, viruses with the highest HA titer were pooled for use in subsequent passages. In addition, viruses passaged 2 or 3 times were propagated in each host to obtain working stocks for this study (A/N-CK or A/N-E). These passaging techniques were described in a manual from World Health Organisation (WHO) Global Influenza Surveillance Network [13].

**Table 1:** GenBank accession numbers of A/Narita/1/2009.

Gene	A/N (cell-isolated)	A/N (egg-isolated)
HA	GQ165814	GQ165815
M	GQ169302	―
NA	GQ166204	GQ166205
NP	GQ169303	―
NS	GQ169304	―
PA	GQ169305	―
PB1	GQ169306	―
PB2	GQ169307	―

### Serial passages of A/N in mice

Cell-isolated A/N from passage 2 (CK2) was used for subsequent serial passages in mice. Briefly, three 6–8-week-old female BALB/c mice (Japan SLC, City of Hamamatsu, Shizuoka Pref.) were inoculated intranasally with 20 μL of culture supernatant containing 7.6 × 10^5^ plaque forming units (pfu) of virus under anesthesia. Three days after infection, the mice were sacrificed and their lungs were washed twice by injecting a total of 2 mL PBS containing 0.1% bovine serum albumin (BSA) [14] as a bronchoalveolar lavage (BAL). The BAL was used for virus titration after removing the cellular debris by centrifugation. BALs from three mice were pooled and used to infect the next group of mice. After 15 passages in mice, the BALs from three mice were pooled and diluted 10-fold before injection into eggs to obtain working stock (A/N-M15). This propagation was used to characterize mice-passaged A/N. In addition, no amino acid substitutions were noted after this final propagation of the virus in eggs (data not shown).

### Virus growth kinetics

To determine the growth kinetics of the different viral passages in three different hosts, viruses were adjusted to a concentration of 1 × HA/mL in PBS. HA titration was performed with 0.5% turkey red blood cells as described by the WHO manual cited earlier [13]. A virus suspension (200 μL) was inoculated into 10-day-old embryonated hens’ eggs (two eggs per virus per time point), or 2 mL of opti-MEM containing 20 μL of virus suspension (0.01 HA/mL) was added to MDCK cells cultured in 6-well plates. At fixed time intervals, eggs were chilled at -80°C for 20 min before samples of allantoic fluid were taken. Samples of MDCK cell culture supernatant were harvested at the same time points. An HA assay was performed to determine viral concentrations in allantoic fluid and culture supernatants for subsequent assessment of virus growth as described by the WHO [13].

### Hemagglutination assay and hemagglutination inhibition assay

A hemagglutination assay and hemagglutination inhibition assay (HAI) was performed with 0.5% turkey red blood cells (TRBC) using the standard method [13]. Turkey whole blood was purchased from Nippon Bio-Test Laboratories, Inc. Ferrets antisera were tested for their capacity to prevent hemagglutination of TRBC induced by various passages of the virus in the different hosts.

### Virus titration

The virus titer in all viral suspensions was determined with a plaque assay or TCID_50_ as described previously [15]. TCID_50_/mL was calculated for each sample using the Reed-Muench method [16]. The virus titer (plaque forming units; PFU) was measured according to the method of Tobita *et al* [17]. Briefly, 200 μl aliquots of serial 10-fold dilutions of the nasal wash were inoculated into MDCK cells in a six-well plate. After allowing the plates to incubate for 1 h, each well was overlaid with 2 ml of agar medium. The number of plaques was counted following crystal violet staining 2 days after inoculation. The virus titer was calculated as pfu per ml (pfu/ml).

### Lethality in mice

To determine the 50% mouse lethal dose (MLD_50_), groups of ten BALB/c mice were inoculated intranasally with 20 μl of 10-fold serial dilutions of A/N in PBS. After a 21-day observation period, the MLD_50_ values were calculated (as pfu) using the Reed-Muench method [16]. After inoculation of the virus into the lungs, mice were monitored every other day for severe disease and more than 25% weight loss. In the present study, symptoms categorized a severe disease were respiratory distress, convulsions, and marked hypoactivity. Euthanasia by exsanguination under anesthesia was performed for mice displaying severe disease or marked weight loss. The humane endpoint was used for all mice meeting the criteria for severe disease or more than 25% weight loss.

### Viral RNA isolation, cDNA amplification, and sequencing

Viral RNA isolation, cDNA amplification, and sequencing of viral HA were performed according to the NITE/NIID Protocol for Sequencing Influenza A (H1N1) SWL Viral Genome Segments [18].

### Statistical analysis

Statistical analysis was performed using the GraphPad Prism statistical software package (Version 5.0c: Graph Pad Software Inc., CA, USA). Comparisons between more than three groups were performed using non-parametric one-way analysis of variance (ANOVA) (Kruskal-Wallis test) with Dunn’s multiple comparison tests. Data were considered statistically significant if the *p* value was less than 0.05.

## Results

### Alteration of virus titer upon serial passaging of A/N in MDCK cells, chicken eggs, or mice

**Fig 1:**
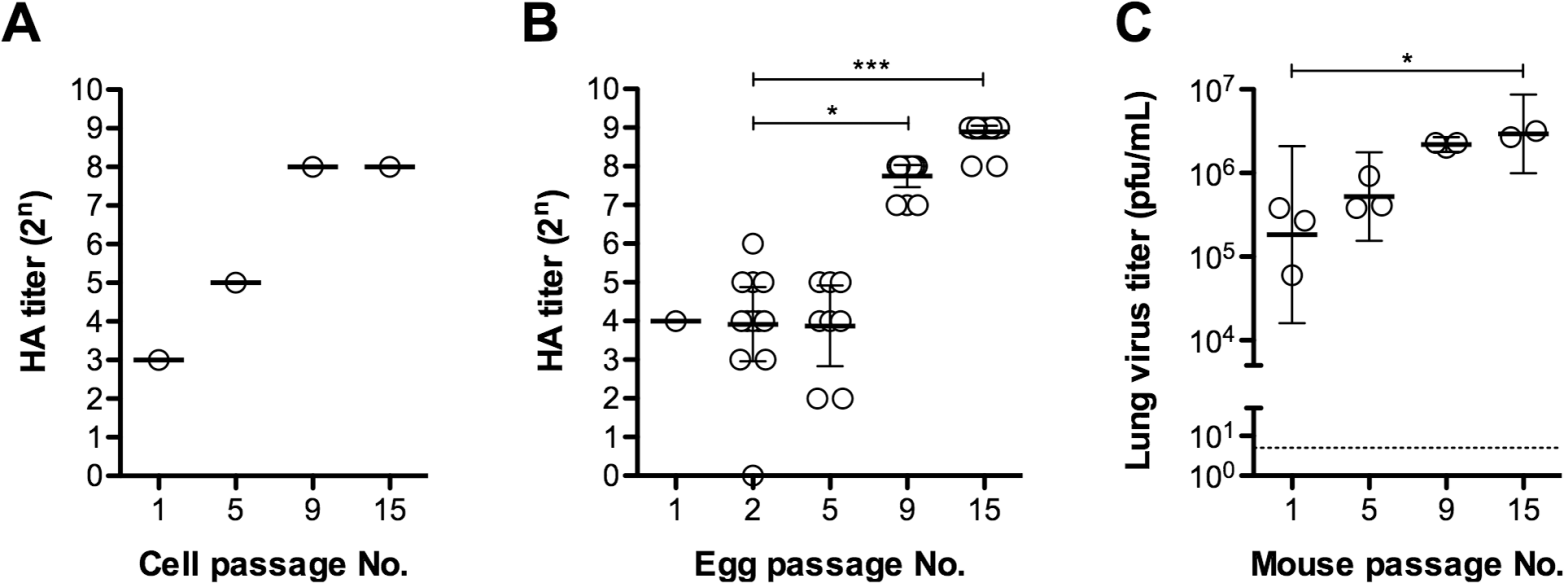
Growth potencial of A/N after multiple passages in cells, eggs or mice. (A) Hemagglutinin (HA) titers of A/N that was both isolated and passaged in cells were determined from the 1^st^, 5^th^, 9^th^, and 15^th^ passages. HA titers in each well of the 6-well culture plate were similar. Therefore, wells were pooled and values represent the HA titer of the pooled wells. (B) HA titers of A/N that was isolated and passaged in eggs (n = 8–18, except for isolation) were determined from the 1^st^, 2^nd^, 5^th^, 9^th^, and 15^th^ passages. Statistical analysis of the 2^nd^ passage was performed. Asterisks * and *** indicate p < 0.05 and p < 0.001, respectively. (C) Mice were infected with cell-isolated A/N and lung washes were collected and used to infect the next group of mice. Lung virus titers of mouse-passaged A/N were determined from the 1^st^, 5^th^, 9^th^, and 15^th^ passages. Statistical analysis of the 1^st^ passage was performed to ascertain adaptation in mouse lungs. The asterisk * indicates *p* < 0.05.

Alterations in the virus titer induced by passaging of the virus in cells, eggs, or mice were analyzed (Fig 1). HA titers of the primary isolated virus (1st passage), and the 5^th^, 9^th^ and 15^th^ passages in cell cultures or eggs were assessed (Fig 1A and 1B), as were the virus titers of the 1^st^, 5^th^, 9^th^ and 15^th^ passages in mice (Fig 1C). The HA titer of both cell- and egg-passaged A/N gradually increased as the passage number increased (Fig 1A and 1B). HA titers of allantoic fluid varied between isolates from the same passage as well as between passages. HA titers ranged < 1–128 (2^7^) from the first inoculation until the 8^th^ egg passage. Upon the 9^th^ egg passage, however, almost all allantoic fluid samples displayed an HA titer of 256 (2^8^) (data not shown). When A/N was passaged in mice, the virus titer of BAL similarly increased gradually and BAL isolated after the 15^th^ passage displayed a titer approximately 10-fold that of BAL obtained after the 1^st^ passage (Fig 1C). These results indicate that prolonged passaging of A/N in cells, eggs, and mice leads to a gradual adaptation to the host, regardless of the type of host.

### Comparison of virus growth kinetics after adaptation to cells, eggs, or mice

**Fig 2:**
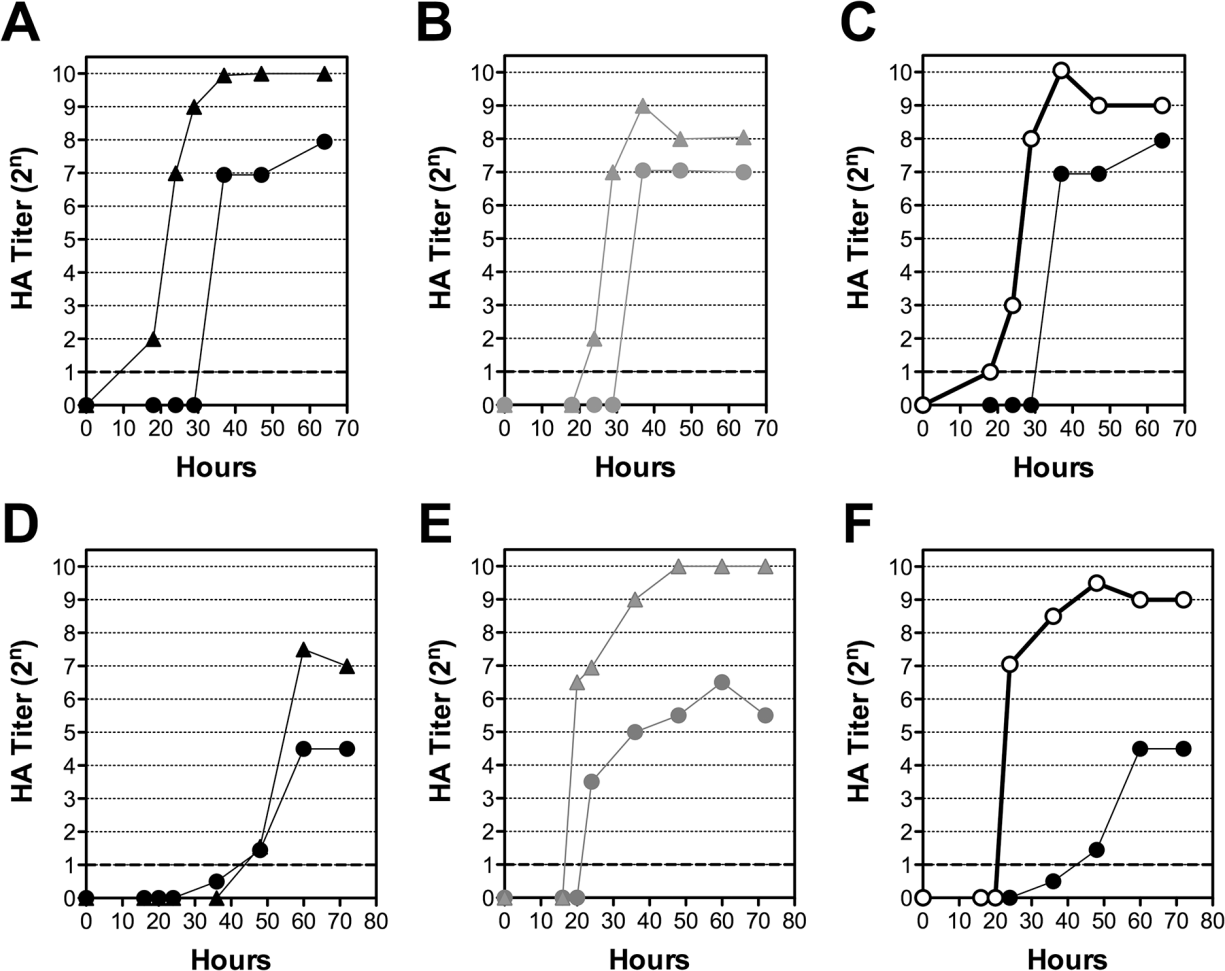
Growth kinetics of A/N in cell cultures or eggs after adaptation to cells, eggs, or mice. Growth kinetics of different viruses in cell culture (A–C) and chicken eggs (D–F). A and D, A/N isolated and passaged 15 times in cells; B and E, A/N isolated and passaged 15 times in eggs; C and F, virus passaged 15 times in mice. For A/N-CK and A/N-E, working stock was used. For details, please see the Materials and Methods.

To clarify the relationship between adaptation of the virus to various hosts and the growth rate in other hosts, the growth kinetics of the viruses that had been subjected to different culture protocols were investigated (Fig 2). A/N that had been grown in MDCK for 15 passages displayed an accelerated and increased growth rate in MDCK cells compared to the virus that had undergone fewer passages in these cells (Fig 2A). Likewise, virus passaged in eggs 15 times also showed an accelerated and increased growth rate in eggs when compared to the virus from earlier passages (Fig 2E). When growth curves of A/N that had been passaged 15 times in eggs or mice were investigated in different hosts, the egg- and mice-adapted viruses also displayed an accelerated and increased growth rate in MDCK cells (Fig 2B and 2C). Although its final titer was higher, A/N that had undergone 15 passages in eggs did not display accelerated growth rate in eggs (Fig 2D). A/N adapted to the murine host showed the greatest increase in growth rate in eggs (Fig 2F).

The infectivity and pathogenicity of A/N passaged in various hosts is summarized in Table 2. Although cell-passaged A/N acquired increased infectivity in cells, no change in infectivity was noted in eggs or mice. In contrast, egg-passaged A/N acquired increased infectivity in both eggs and cells. Similarly, mouse-passaged A/N displayed increased infectivity in both eggs and cells and also exhibited a 20-fold increase in pathogenicity. Thus, the mouse-passaged A/N grew efficiently in both cells and eggs and also had a high level of infectivity in cells and eggs and a high level of lethality in mice.

**Table 2:** Characterization of A/N passaged through different hosts.

Virus*	Virus titer	EID_50_	TCID_50_	MLD_50_ **
	(pfu/mL)	(log_10_EID_50_/mL)	(log_10_TCID_50_/mL)	(pfu/20μL)
A/N-CK	4.4 x 10^7^	4.9	7.4	> 1.0 x 10^5^ ***
A/N-CK15	4.5 x 10^8^	5	8.1	> 1.0 x 10^6^ ***
A/N-E	1.7 x 10^8^	7.8	7.4	1.6 x 10^5^
A/N-E15	1.0 x 10^9^	9.3	8.2	8.0 x 10^5^
A/N-M15	1.2 x 10^9^	8.5	8.9	8.2 x 10^3^

* For A/N-CK, A/N-E and A/N-M15, working stock was used for characterization. For details, please see the Materials and Methods.

** Groups of 10 BALB/c mice inoculated intranasally with 20 μl of 10-fold serial dilutions of A/N under anesthesia.

***Lowest detection limit

### Amino acid substitution and its effects on antigenicity after A/N passaging in cells, eggs, or mice

The genomes of A/N passaged in cells, eggs, or mice were analyzed to investigate whether adaptation to different hosts was accompanied by amino acid substitutions. Amino acid substitutions are summarized in Table 3. Changes in amino acid were noted in HA in cells passaged through cells, eggs, and mice. In A/N passaged in cells 15 times, the amino acid at position 155 changed from glycine (G) to glutamic acid (E). In addition, a mixture of threonine (T) and asparagine (N) was found at position 355. In A/N passaged 15 times in eggs, glutamine (Q) at position 223 was substituted with arginine (R). Two substitutions were noted in the virus from mouse lung wash samples obtained after 15 passages. These substitutions, at positions 156 (N156D) and 222 (D222G), were not noted in viruses passaged through eggs or cells. In addition, these two alterations of A/N passaged 15 times in mice persisted for at least two cycles when the virus was cultured in the allantoic fluid of eggs. Amino acid changes were noted within other proteins: K331E in NA was noted in A/N-CK15; F53V in PA, D101N in NP, and M165I in M1 were noted in A/N-E15; and E158G in PB2 was noted in A/N-M15.

**Table 3:** Amino acid substitutions in cell-, egg- or mouse-passaged A/N*.

	Amino acid substitutions**					
Antigen***	HA	NA	PB2	PA	NP	M1
A/N-CK	―	―	―	―	―	―
A/N-CK15	G155E N355N/T	K331E	―	―	―	―
A/N-E	―	―	―	―	―	―
A/N-E15	G155E Q223R	―	―	F53V	D101N	M165I
A/N-M15	N156D D222G	―	E158G	―	―	―

* The amino acid number denotes the position in the sequence of the H1 subtype.

** No amino acid substitution was noted in PB1 or NS.

*** For A/N-CK, A/N-E and A/N-M15, working stock was used for test antigen. For details, please see the Materials and Methods.

Whether A/N changed its antigenic specificity as a result of the HA mutations acquired during the course of cell, egg and mouse passaging was investigated next. The antigenic properties of parental and various host-passaged A/N variants were determined in an HAI test using post-infection ferret antisera (Table 4). A/N-E reacted strongly with the ferret anti-A/N-E sera and reacted strongly with anti-A/N-CK sera. The viruses that were passaged 15 times, irrespective of the host, displayed a reduced ability to react with ferret antisera against both A/N-CK and A/N-E. However, the adapted viruses reacted more strongly to ferret antisera against the cell-passaged A/N (4-fold difference) than they did to ferret antisera against the egg-passaged A/N (10-fold difference). These results suggest that, in contrast to the egg- and mouse-passaged virus, the cell-passaged A/N proliferated efficiently while retaining its antigenic and pathogenic properties.

**Table 4:** HAI assay of A/N with ferret antisera.

	Geometric mean titer of HAI titer**	
Test antigen*	Ferret anti-serum against A/N-CK	Ferret anti-serum against A/N-E
A/N-CK	3225	4064
A/N-CK15	806	320***
A/N-E	2560	3225
A/N-E15	403***	202***
A/N-M15	403***	320***

* For A/N-CK, A/N-E and A/N-M15, working stock was used for test antigen. For details, please see the Materials and Methods.

** The homologous titer to respective sera designated in this study is underlined.

*** The HAI titer in bold reflects an 8-fold difference with respect to the homologous titer.

## Discussion

This study characterized the proliferative capacity, and pathogenicity of antigenic changes in A/N, the first H1pdm strain to be isolated in Japan, after adaptation to MDCK cells, chicken eggs, and mice. Most influenza viruses acquired amino acid substitutions in HA upon prolonged passaging in eggs, and this finding warrants consideration during vaccine manufacture. The H1pdm strain X-179A was selected for use in the 2010 seasonal vaccine in Japan. H1pdm viruses generally grow poorly in embryonated chicken eggs, and classical reassortant technology was therefore used on an A/California/7/2009 virus at New York Medical College in 2009 to produce strain X-179A [7].

The growth rate of A/N was found to change upon prolonged passaging in cells, eggs, and mice (Fig 1). With each serial passage in cells and eggs, the harvested viruses were diluted a thousand times before inoculation in the next series of cells and eggs, and BAL fluid was transferred to the next generation of mice without dilution. Therefore, the virus suspensions used for inoculation contained increasing doses of virus with every passage. Under these passaging conditions, A/N passaged 15 times had a higher HA titer than A/N passaged fewer times (Fig 1). When the same MOI was used in passaging, A/N passaged 15 times had a higher HA titer than A/N passaged fewer times (data not shown). In light of these results, the increased HA titer indicates that cell- and egg-passaged A/N had a higher growth rate as a result of adaptation, regardless of the virus dose administered. Mouse-passaged A/N was predicted to have and increased virus titer like cell- and egg-passaged A/N. Moreover, prolonged passaging in one host may positively affect the growth rate in other hosts. Virus growth kinetics and changes to the amount of virus that could be harvested after each passagewere also analyzed. In increased growth rate or pathogenicity of influenza viruses after prolonged passaging can be attributed to amino acid substitutions [19], and amino acid changes were therefore monitored in the current study. Amino acid changes were observed at positions 155 and 355 (within HA) after prolonged passaging in cells, and these changes were accompanied by a low HAI titers. However, the amino acid at position 355 is situated within the HA2 domain, which is not determined in the HAI assay. Therefore, low HAI titers were solely caused by the amino acid substitution at position 155. However, the adapted viruses reacted more strongly to ferret antisera against the cell-passaged A/N (4-fold difference) than to ferret antisera against the egg-passaged A/N (10-fold difference) (Table 4). These differences in the reduction of HAI may be caused by the different hosts that were used for virus isolation. As this study has shown, prolonged passaging in cells or eggs resulted in different substitutions. Therefore, virus isolates from each host presumably already had undetectable genetic variants, and these variants caused different HAI reactions to antigens. In addition, the K331E was observed in NA after prolonged passaging in cells. Two strains of K331E at NA were also identified from among 135 of clinical isolates from Finnish patients (accession nos. KF559520 and KM366353) [20]. There is no current evidence of this substitution having a specific effect, but its effect of ths substitution on the phenotype of the viral population should be investigated.

At present, influenza vaccines are largely manufactured using chicken eggs, but a cell culture-based seasonal influenza vaccine is likely to be approved in Japan in the near future. There are several advantages of cell-based manufacturing over current techniques that use embryonated chicken eggs to grow the virus [21–23]. First, obtaining sufficient eggs can be challenging when large amounts of vaccine need to be produced in a short period of time, as in the case of a pandemic; the use of a cell-based manufacturing system circumvents this requirement (http://www.cdc.gov/flu/protect/vaccine/cell-based.htm). Second, the use of cells for production allows vaccines to be used by individuals with egg allergies, and third, cell-based manufacture avoids the selection of egg-adapted variants with altered virus antigenicity [24]. However, the current results indicate that cell-passaging of influenza H1pdm strains can induce amino acid substitutions at positions 153 to 157 that are similar to the changes found upon viral adaptation to eggs. These changes can lead to a marked reduction in the ability to react with ferret antisera [25]. The current study also noted an amino acid substitution at position 223 after passaging in chicken eggs, and this substitution that had been noted previously [26]. Earlier research found that this substitution did not affect responses in an HAI assay even though the substitution affected the receptor-binding site [11,25]. A/California/7/2009, the original strain from which the X-179A H1pdm vaccine strain was derived, has glutamine (Q) at position 223 of HA. By contrast, X-179A has arginine (R) at this position. This was induced by passaging in eggs, but was not found to affect the HAI titer. Thus changes in antigenicity upon prolonged passaging are a risk not only in egg-based but also in cell-based vaccine production.

After prolonged passaging in mice, A/N developed a high level of pathogenicity in mice and exhibited faster growth in cells and eggs; however, low HAI titers were detected when using ferret antisera. Ilyushina *et al*. investigated the adaptation of H1pdm, A/California/04/2009 (A/CA/04/09) and A/Tennessee/1-560/2009 (A/TN/1-560/09), to BALB/c mice and noted amino acid substitutions in the HA protein at positions 155, 183, and 222 for A/CA/04/09 and at positions 119 and 221 for A/TN/1-560/09 [27]. Both strains were found to be significantly more virulent in mice than their respective parental viruses, despite the distinct amino acid changes in the two adapted strains. A/CA/04/09 had a reduced HAI titer after passages in mice, whereas A/TN/1-560/09 reacted more strongly with ferret antisera. In the current study, A/N exhibited amino acid substitutions in the HA protein at positions 156 and 222 after passaging in mice. This resembled changes in A/CA/04/09 that resulted in a decreased HAI titer. However, changes in antigenicity were caused by amino acid substitution at position 156 on HA [25], so there was no evidence of a causal relationship between the substitution at position 222 and a reduced HAI titer. Another study found that the E158G substitution in PB2 led to increased morbidity and mortality in mice [28]. The current study also noted a substitution at position 158 in PB2 in A/N passaged in mice. The likely conclusion is that this amino acid substitution within PB2 caused the high level of pathogenicity in mice. In addition, amino acid changes in other proteins, i.e., F53V in PA, D101N in NP, and M165I in M1 were noted in A/N-E15, but there is no evidence that substitutions induced phenotypic changes.

This study investigated the effects of passaging A/N multiple times in MDCK cells, chicken eggs, and mice. Results indicated that the virus adapted to these three different hosts and that this adaptation led to an increase in the virus titer. Moreover, viral growth rates increased after viral adaptation to the host in moset instances. The virus that had adapted to eggs or mice also had an increased growth rate in cell culture. The genetic alterations underlying adaptive changes were also ascertained. Taken together, these findings indicate that different virus strains adapt to hosts by similar amino acid substitutions and that viral adaptation occurs in both cell-based and egg-based culture.
